# Ilheus Virus Isolation in the Pantanal, West-Central Brazil

**DOI:** 10.1371/journal.pntd.0002318

**Published:** 2013-07-18

**Authors:** Alex Pauvolid-Corrêa, Joan L. Kenney, Dinair Couto-Lima, Zilca M. S. Campos, Hermann G. Schatzmayr, Rita M. R. Nogueira, Aaron C. Brault, Nicholas Komar

**Affiliations:** 1 Laboratório de Flavivírus, Instituto Oswaldo Cruz, Fundação Oswaldo Cruz, Ministério da Saúde, Rio de Janeiro, Rio de Janeiro, Brasil; 2 Arbovirus Diseases Branch, Centers for Disease Control and Prevention, Fort Collins, Colorado, United States of America; 3 Fulbright Visiting Researcher in Doctorate Sandwich Program at CDC, Fort Collins, Colorado, United States of America; 4 Embrapa Pantanal, Corumbá, Mato Grosso do Sul, Brasil; United States Army Medical Research Institute of Infectious Diseases, United States of America

## Abstract

The wetlands of the Brazilian Pantanal host large concentrations of diverse wildlife species and hematophagous arthropods, conditions that favor the circulation of zoonotic arboviruses. A recent study from the Nhecolândia sub-region of Pantanal reported serological evidence of various flaviviruses, including West Nile virus and Ilheus virus (ILHV). According to the age of seropositive horses, at least three flaviviruses, including ILHV, circulated in the Brazilian Pantanal between 2005 and 2009. To extend this study, we collected 3,234 adult mosquitoes of 16 species during 2009 and 2010 in the same sub-region. Mosquito pool homogenates were assayed for infectious virus on C6/36 and Vero cell monolayers and also tested for flaviviral RNA by a group-specific real-time RT-PCR. One pool containing 50 non-engorged female specimens of *Aedes scapularis* tested positive for ILHV by culture and for ILHV RNA by real-time RT-PCR, indicating a minimum infection rate of 2.5 per 1000. Full-length genomic sequence exhibited 95% identity to the only full genome sequence available for ILHV. The present data confirm the circulation of ILHV in the Brazilian Pantanal.

## Author Summary

Ilheus virus causes mainly asymptomatic infections in humans with rare reports of encephalitis in northern South America. Ilheus virus is believed to be maintained in zoonotic cycles between birds and mosquitoes in Central and South America. Aiming to detect Ilheus virus and other arboviruses in the Pantanal region of west-central Brazil, host-seeking mosquitoes were collected by aspiration as they landed on horses, research team members, and caimans. Resting mosquitoes were collected from the walls inside a local residence and additional flying mosquitoes were captured in light traps. We isolated a Brazilian strain of Ilheus virus from a pool of 50 *Aedes scapularis* mosquitoes that were collected while landing on a member of our research team, in April, 2010. Our finding confirms a recent report of Ilheus virus activity in the Pantanal derived from the detection of neutralizing antibodies in horses. The anthropophilic behavior of the infected mosquito(es) suggests a risk of Ilheus virus transmission from mosquitoes to humans in the region. We provide the virus' complete genomic sequence and present its phylogenetic relationship with other isolates.

## Introduction

Ilheus virus (ILHV) was first described in 1944, when it was isolated from *Aedes* and *Psorophora* spp. mosquitoes during an epidemiological investigation of yellow fever in the city of Ilhéus, state of Bahia, northeast Brazil [1], [2]. According to the Ninth Report of the International Committee on Taxonomy of Viruses, ILHV is included in the Ntaya virus group [3]. ILHV causes mainly asymptomatic infections in humans with rare reports of encephalitis throughout northern South America [4], [5]. Human infection with ILHV has been reported in Trinidad, Panama, Colombia, French Guyana, Brazil, Ecuador and Bolivia [6], [7], [8], [9], [10], [11], [12].

ILHV is believed to be maintained in zoonotic cycles between birds and mosquitoes and has been isolated in Central and South America primarily from mosquitoes [13], [14], [15], [16]. ILHV has been previously isolated from eight mosquito genera, but also from sentinel monkeys, man and birds. In antibody surveys, neutralizing antibodies for ILHV have been found in rodents, birds, sentinel monkeys and man [17]. In the 1990s, ILHV was isolated from wild birds in southeast Brazil [18].

Little is known about ILHV infections in domestic animals. In the 1990s, a serological investigation conducted among equines from the Pantanal region of west-central Brazil found neutralizing antibodies to ILHV suggesting its circulation in the region [19]. In 2009, a flavivirus serosurvey conducted in the same area among equines from the Nhecolândia sub-region of the Pantanal detected evidence of past infection with, in increasing order of seroprevalence, Cacipacore virus (CPCV), West Nile virus (WNV), Saint Louis encephalitis virus (SLEV) and ILHV [20].

The Brazilian Pantanal is a seasonally flooded surface of approximately 140,000 km^2^. The area is divided into 11 sub-regions based on characteristics of seasonal floods, physiography and ecology. Nhecolândia is the second largest of these sub-regions with approximately 27,000 km^2^ and the world's largest and most diverse area of subtropical lakes encompassing approximately 10,000 lakes [21], [22], and a human population of less than 22,500 in 2010 [23].

The wetlands of the Brazilian Pantanal host large concentrations of diverse wildlife species and hematophagous arthropods, conditions that favor the circulation of zoonotic arboviruses. In spite of scarce data concerning arbovirus vectors in the area, the local movement of mosquito species previously reported as arbovirus vectors has been reported in the north and south of Pantanal [24], [25].

Serological surveys conducted in local horses have found neutralizing antibodies for at least eight arboviruses, including the bunyaviruses Tacaiuma virus and Maguari virus, the alphaviruses eastern equine encephalitis virus and western equine encephalitis virus and the flaviviruses CPCV, SLEV, WNV and ILHV [19], [20], [26], [27].

Despite the recent detection of neutralizing antibodies to various flaviviruses in horses from the Nhecolândia sub-region, circulation of these arboviruses in this area was never confirmed by virus isolation. Therefore, in an effort to detect flaviviruses currently being transmitted in this region, we collected and tested adult female mosquitoes for infectious virus and for flavivirus RNA detection.

## Materials and Methods

### Study area

**Figure 1:**
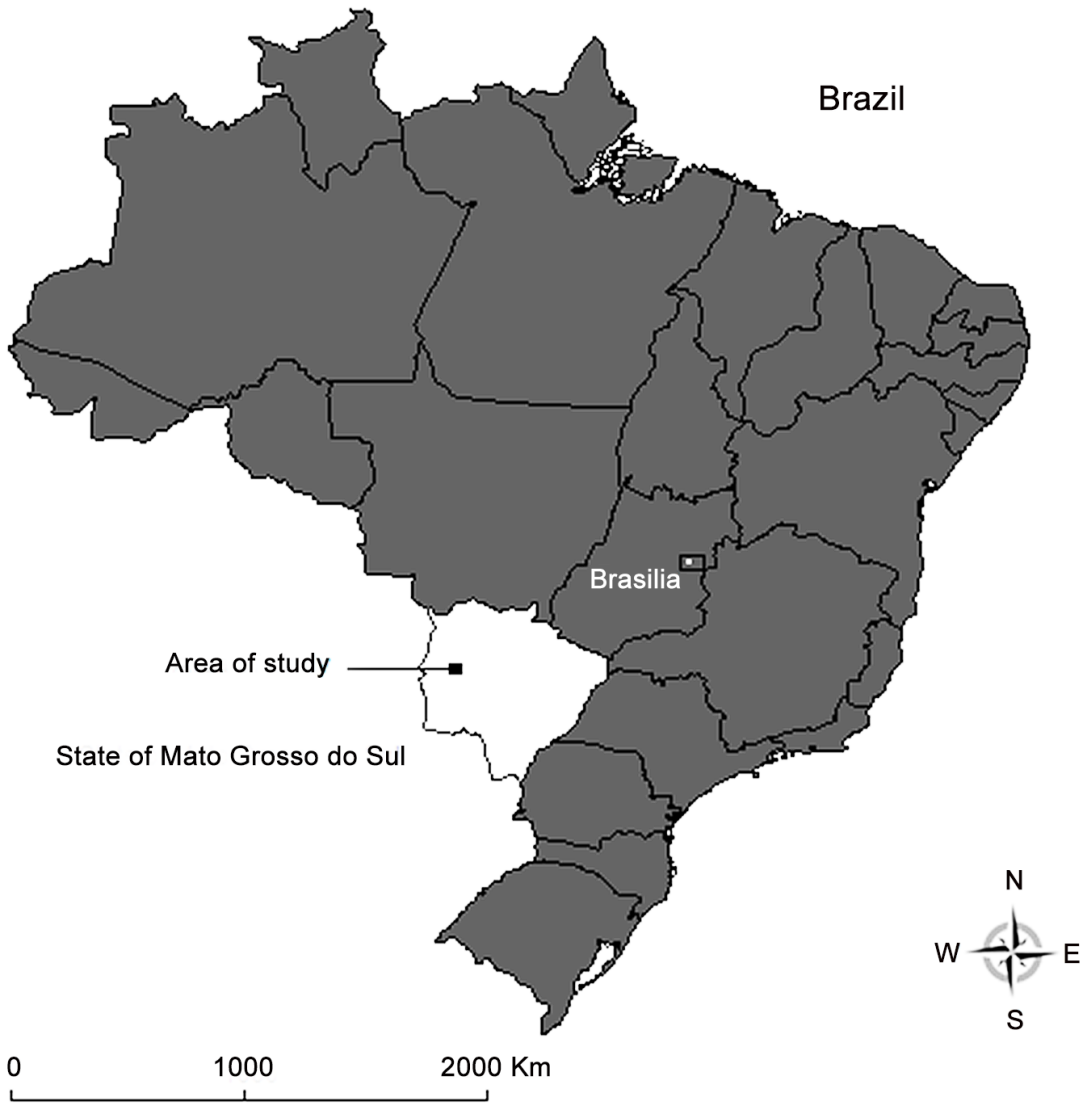
Area of study in the Pantanal, State of Mato Grosso do Sul, Brazil.

In October 2009, April and October 2010, attempts to collect adult mosquitoes were undertaken at sites randomly selected within eight beef cattle ranches comprising a large area of approximately 1,500 km^2^ in the Nhecolândia sub-region of Pantanal, west-central Brazil (18°18′–19°15′S, 57°05′–55°24′W) (Figure 1). The collections for this study were authorized by the owners or residents of the sampled properties and also by the Animal Ethics Committee of Fundação Oswaldo Cruz (License CEUA-Fiocruz LW-1/12, protocol P-74/10-5) and the Brazilian Institute of Environment and Natural Resources (licenses IBAMA 18363-1/2009 and 18363-2/2010).

### Mosquito collections

A total of 50 attempts to collect adult mosquitoes were undertaken, but only 39 were positive for mosquitoes. From 11 unsuccessful attempts, five were undertaken in October 2009 and six in October 2010, during the local dry season. Among the 39 positive collections, 12 were undertaken in October 2009, 15 in April 2010 and 12 in October 2010. Seventeen collections were conducted using CDC light traps, six collections using manual aspirators from horses, ten on research team members, three on caimans and three using manual aspirators while landing on walls inside a local residence. Mosquitoes were transported alive to a field laboratory where they were immobilized by cold anesthesia and were identified by species based on morphologic appearance according to taxonomic keys [28], [29]. The mosquitoes were grouped into pools of up to 50 individuals that were sorted with respect to collection method, site of capture, species and gender and then stored in liquid nitrogen.

### Virus isolation

Mosquito samples were assayed for viral cytopathic effect (CPE) on C6/36 and plaque formation on Vero cell cultures using standard protocols [30]. Briefly, pools from one up to 50 mosquito specimens were placed in polypropylene capped 2 mL tubes with copper-clad steel beads and 1 mL of BA-1 diluent (1× Medium 199-H, 0.05 M TRIS-HCI, 1% Bovine serum albumin, 4 mM L-Glutamine, 0.35 g/L Sodium bicarbonate, 200 U/mL Penicillin, 200 µg/mL Streptomycin, 2 µg/mL Amphotericin B and 50 µg/mL Gentamycin). A 0.1 mL aliquot of each triturated mosquito pool was inoculated in duplicate onto C6/36 and Vero monolayers prepared in 6-well polystyrene culture plates. The plates were maintained at 28°C and 37°C, respectively, for 1 h with discrete motion every 15 min to optimize virus adsorption. At the end of this period, 3 mL of maintenance media (1× DMEM, Sterile water, 2% Fetal bovine serum, 7.5% Sodium bicarbonate, 1 M HEPES, Non-essentials amino acids, Sodium pyruvate, 200 U/mL Penicillin, 200 µg/mL Streptomycin, 2 µg/mL Amphotericin B and 50 µg/mL Gentamycin) were added to the C6/36 cultures and 3 mL of 0.5% agarose in overlay media with 2× Ye-Lah overlay medium (10× Earle's BBS, Ye-Lah medium, 2% Fetal bovine serum and Sterile distilled water), 7.5% Sodium bicarbonate, 200 U/mL Penicillin, 200 µg/mL Streptomycin, 2 µg/mL Amphotericin B and 50 µg/mL Gentamycin) were added to the Vero cultures, respectively. Plates with C6/36 monolayers were incubated at 28°C in 5% CO_2_ for 10 days, and observed daily for viral CPE. Plates with Vero monolayers were divided in two groups and then incubated at 37°C at 5% CO_2_. The first group was incubated for two days and the second group for five days before being stained with neutral red in 3 mL of 0.5% agarose culture media. Both groups were kept at 37°C in 5% CO_2_ for 10 days, and observed daily for viral plaques.

### Real-time RT-PCR

A Sybr green real-time RT-PCR method for the universal detection of flaviviruses was performed to test all mosquito samples for flaviviral nucleic acid. First, RNA was extracted from triturated mosquitoes, and then tested for flavivirus genus-specific viral RNA by real-time RT-PCR using the QiaAmp viral RNA kit (Qiagen, Valencia, CA, USA). The protocol chosen is based on the amplification of a 269–272 nucleotide region at the N terminus of the NS5 gene [31]. Amplicons of samples that presented a melting curve peak above 90 RFU at a temperature above 75°C were considered positive and selected for purification and cDNA sequencing using both forward and reverse primers in separate reactions. DNA amplifications and sequencing reactions were completed on a DNA Engine PTC-200 thermal cycler (Bio-Rad Laboratories, Hercules, CA, USA) and analyzed on an ABI 3130 genetic analyzer (Applied Byosystems, Foster City, CA, USA). Sequences were assembled using Lasergene 9 software (DNA STAR, Madison, WI, USA) and subjected to a BLASTn analysis.

### Full genome sequencing and phylogenetics

Amplification of total RNA was performed utilizing the SuperScript III one-step RT-PCR kit (Invitrogen, Carlsbad, CA, USA) and universal NS5 specific flavivirus primers (cFD3 and FU1) [32]. An initial fragment of approximately 1,000 base pairs was generated and subjected to GenBank BLAST analysis for identification. Once initial sequencing of the NS5 region indicated high identity with ILHV, primers for full-length sequencing were designed utilizing Primer 3 software in Geneious R6 based on the full-length ILHV sequence in GenBank (NC_009028). Fragments were extracted from an agarose gel with the Qiagen Qiaquick kit and subjected to nucleotide sequencing using BigDye Terminator V3.1 Cycle Sequencing kit (Applied Biosystems, Foster City, CA, USA) on a DNA Engine PTC-200 thermal cycler (Bio-Rad Laboratories, Hercules, CA, USA). The products were purified using BigDye Xterminator product (Applied Biosystems, Foster City, CA, USA) and then subjected to an ABI 3130 Genetic Analyzer (Applied Byosystems, Foster City, CA, USA). Sequencing files were aligned and analyzed utilizing Geneious R6 software. The full-length sequence was aligned with homologous flavivirus genomes utilizing MAFFT v7.017 [33], and trees analyzing the full genome sequence as well as the individual NS5 and E genes were generated using maximum-likelihood analyses with a PhyML algorithm in SeaView 4.0 [34] with 1,000 bootstrap replicates. Genome sequences of all flaviviruses sequences utilized are listed in GenBank under the following NCBI Reference Sequences: Aedes flavivirus (NC_012932); Kamiti River virus (NC_005064); Cell fusing agent virus (NC_001564); Culex flavivirus (NC_008604); Wesselsbron virus (NC_012735); yellow fever virus (NC_002031); dengue virus 4 (NC_002640); dengue virus 2 (NC_001474); dengue virus 1 (NC_001477); dengue virus 3 (NC_001475); Kedougou virus (NC_012533); Zika virus (NC_012532); Aroa virus (NC_009026); West Nile virus (NC_009942); Japanese encephalitis virus (NC_001437); Murray Valley encephalitis virus (NC_000943); Usutu virus (NC_006551); St. Louis encephalitis virus (NC_007580); Bagaza virus (NC_012534); Ilheus virus (NC_009028); Powassan virus (NC_003687); Alkhurma virus (NC_004355); Langat virus (NC_003690); Omsk hemorrhagic fever virus (NC_005062); tick-borne encephalitis virus (NC_001672); Apoi virus (NC_003676); Modoc virus (NC_003635); Rio Bravo virus (NC_003675).

## Results

A total of 3,234 mosquitoes of at least 16 species were collected from five out of the eight ranches where mosquito collection attempts were undertaken (Table 1). Mosquitoes of at least 11 species were identified from human landing collections, 11 from equine landing collections, 11 from CDC light traps, four from caimans and only one indoor.

**Table 1:** Distribution of mosquitoes captured in the Pantanal, Brazil in 2009 and 2010.

	OCTOBER 2009							APRIL 2010									OCTOBER 2010														
	CDC		EQ		H		S-T	CDC			EQ			H		S-T	CDC			EQ			H		C		I			S-T	Total
	NE	E	NE	E	NE	E		NE	E	M	NE	E	M	NE	E		NE	E	M	NE	E	M	NE	E	NE	E	NE	E	M		
*Mansonia* spp.	1						1	44	1		384	259		9	3	700						1								1	702 (21.7%)
*Culex* spp.							0	384	6	101	26	4	1	8		531															531 (16.4%)
*Aedes scapularis*	5		2	3	126	6	142	20	1		61	20		139	7	248							14	2	41	13				70	460 (14.2%)
*Culex quinquefasciatus*	8				2		10	25		1				1		27	50	8	57	1							87	10	118	331	368 (11.4%)
*Mansonia pseudotitillans*	2				15		17									0		1		131	45		5		14					196	213 (6.6%)
*Mansonia titillans*	19		5	1	45		70	13			20			48		81									42	8				50	201 (6.2%)
*Culex chidesteri*							0	146						27	2	175															175 (5.4%)
*Anopheles Nyssorhynchus* spp.	1						1	9	1		25	21		12		68				90										90	159 (4.9%)
*Anopheles Nyssorhynchus argyrotarsis*							0	1			69	23		8		101				38	2									40	141 (4.4%)
*Culex declarator*							0	38			7	2				47															47 (1.5%)
*Psorophora albigenu*	1				32	4	37				1			2		3										5				5	45 (1.4%)
*Anopheles Nyssorhynchus triannulatus s.l.*							0				2					2				36	6									42	44 (1.4%)
*Mansonia amazonenses*					29		29									0	2						7							9	38 (1.2%)
*Mansonia humeralis*							0					2		22		24															24 (0.7%)
*Uranotaenia* spp.	2						2	17		3						20	1													1	23 (0.7%)
*Culex coronator*							0	16								16															16 (0.5%)
*Aedeomyia squamipennis*							0	12		1						13	2													2	15 (0.5%)
*Psorophora* spp.							0	1						8	2	11															11 (0.3%)
*Uranotaenia lowii*							0	7	1							8															8 (0.2%)
*Anopheles Nyssorhynchus albitarsis s.l.*					1		1				6					6															7 (0.2%)
*Psorophora ciliata*			2	1			3							1		1															4 (0.1%)
*Anopheles* sp.							0	1								1															1 (0.03%)
*Aedes stegomya* spp.							0							1		1															1 (0.03%)
Total	39		9	5	250	10	313	734	10	107	601	331	1	286	14	2084	55	9	57	296	53	1	26	2	97	26	87	10	118	837	3234 (100%)

CDC- Outdoors Centers for Disease Control light trap; EQ- Equine landing collections; H- Human landing collections; NE- Non-engorged; E- Engorged; C- Caiman landing collections; I- Indoor collections; M- Male; S-T- Sub-total.

Among the 1,297 specimens caught landing on horses, the most common species was *Mansonia pseudotitillans* (*n = *177; 13,6%). Interestingly, all the specimens were only collected in October 2010, indicating a marked fluctuation of this species in the region. On the other hand, among all different methods of capture used, *Uranotaenia lowii, Cx. coronator, Cx. declarator, Cx. chidesteri and Ma. humeralis* were only detected in the April collections.

Among the mosquitoes identified to species, *Ae. scapularis* was the most prevalent with 460 (14.2%) specimens of all mosquitoes captured. Most were caught while attempting to acquire blood from humans (n = 294; 64%) and horses (n = 87; 18.7%) while only 54 (11.7%) were captured on caimans, 26 (5.7%) in CDC light traps, and none indoors, indicating anthropophilic behavior with no evidence for endophily. *Ae. scapularis* was the most abundant species identified in October 2009 and in April 2010, whereas *Cx. quinquefasciatus* was the most prevalent species among mosquitoes collected in October, 2010.

*Ae. scapularis* tested positive for ILHV by culture and for ILHV RNA by real-time RT-PCR, indicating a minimum infection rate of 2.5 per 1,000. Among the 331 specimens of *Cx. quinquefasciatus* collected in October 2010, 215 (64.9%) were removed from the walls of a local residence, 115 (34.7%) were obtained using CDC light traps and only one (0.3%) specimen was caught while blood-feeding on a horse, confirming its endophilic behavior.

**Figure 2:**
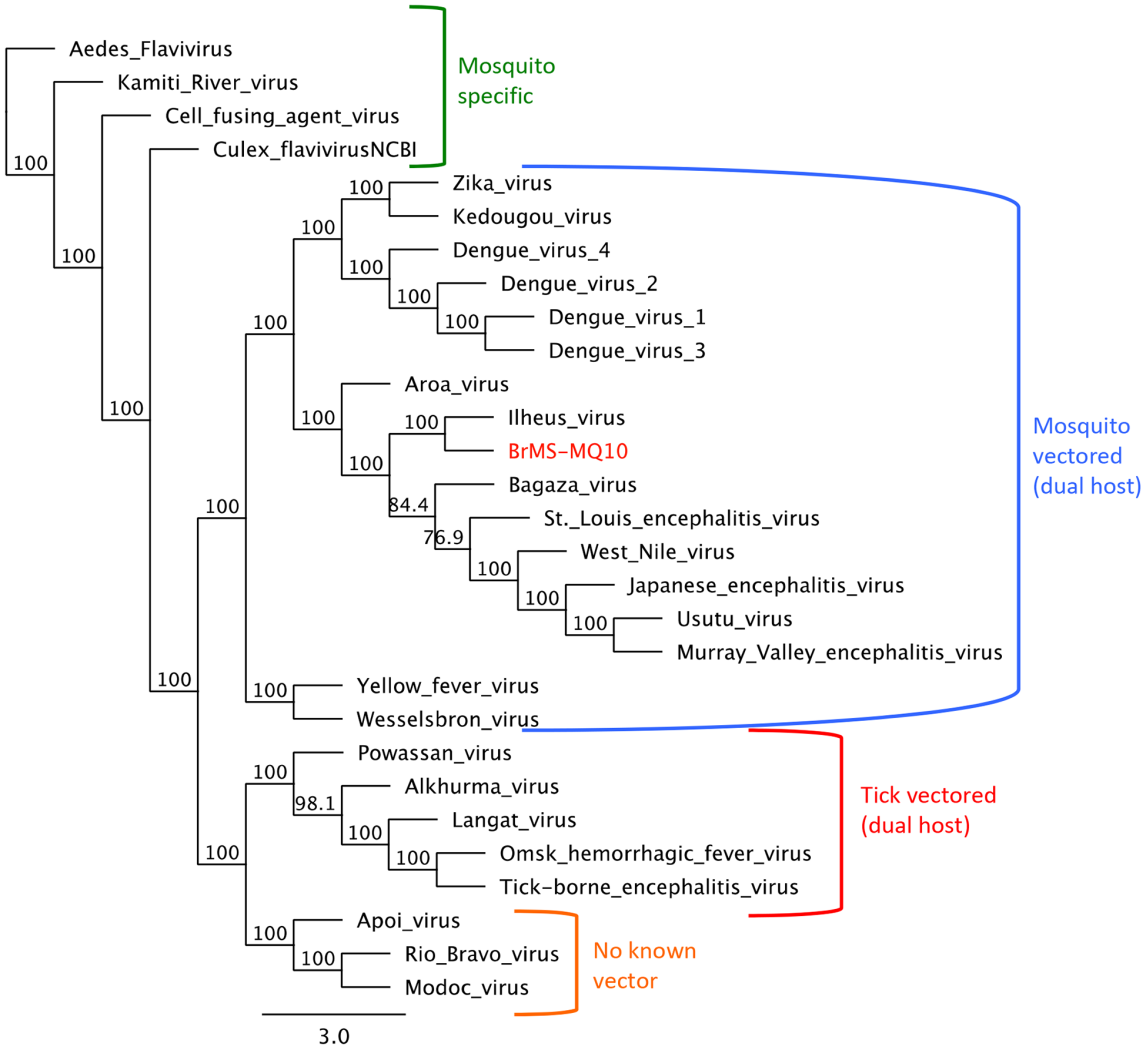
Phylogenetic relationships of full genome of ILHV isolate BrMS-MQ10. Maximum-likelihood tree obtained from full coding sequences using a GTR substitution model. Branches are labeled with bootstrap values that represent the percentage of 1,000 replicates in which the members of a given clade were predicted to relate in the same topography.

**Figure 3:**
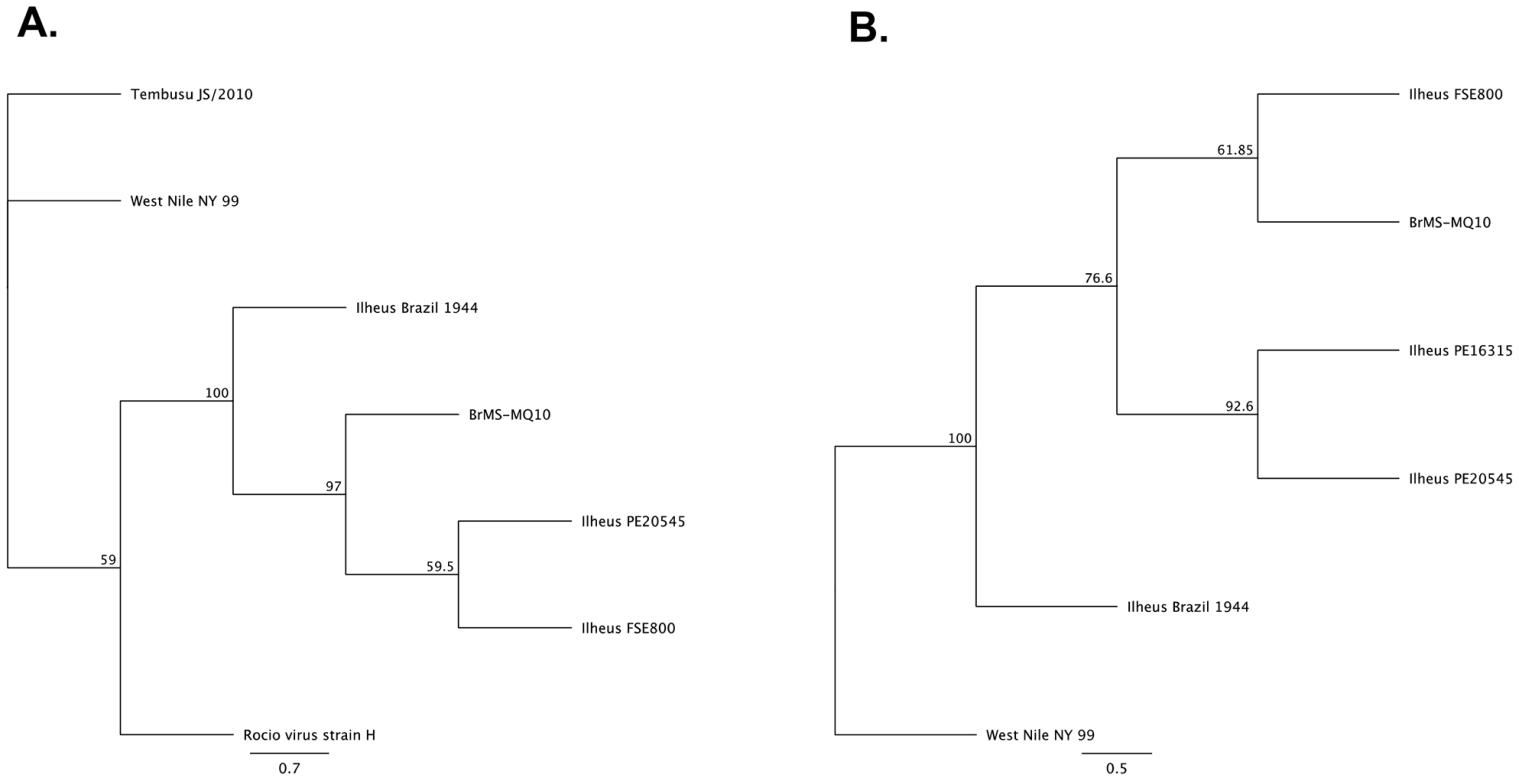
Phylogenetic relationship of NS5 and Envelope genes. Neighbor-joining trees obtained from (A) available NS5 sequences and (B) available Envelope sequences. Percentage above the branches represents bootstrap values from 1,000 replicates.

**Figure 4:**
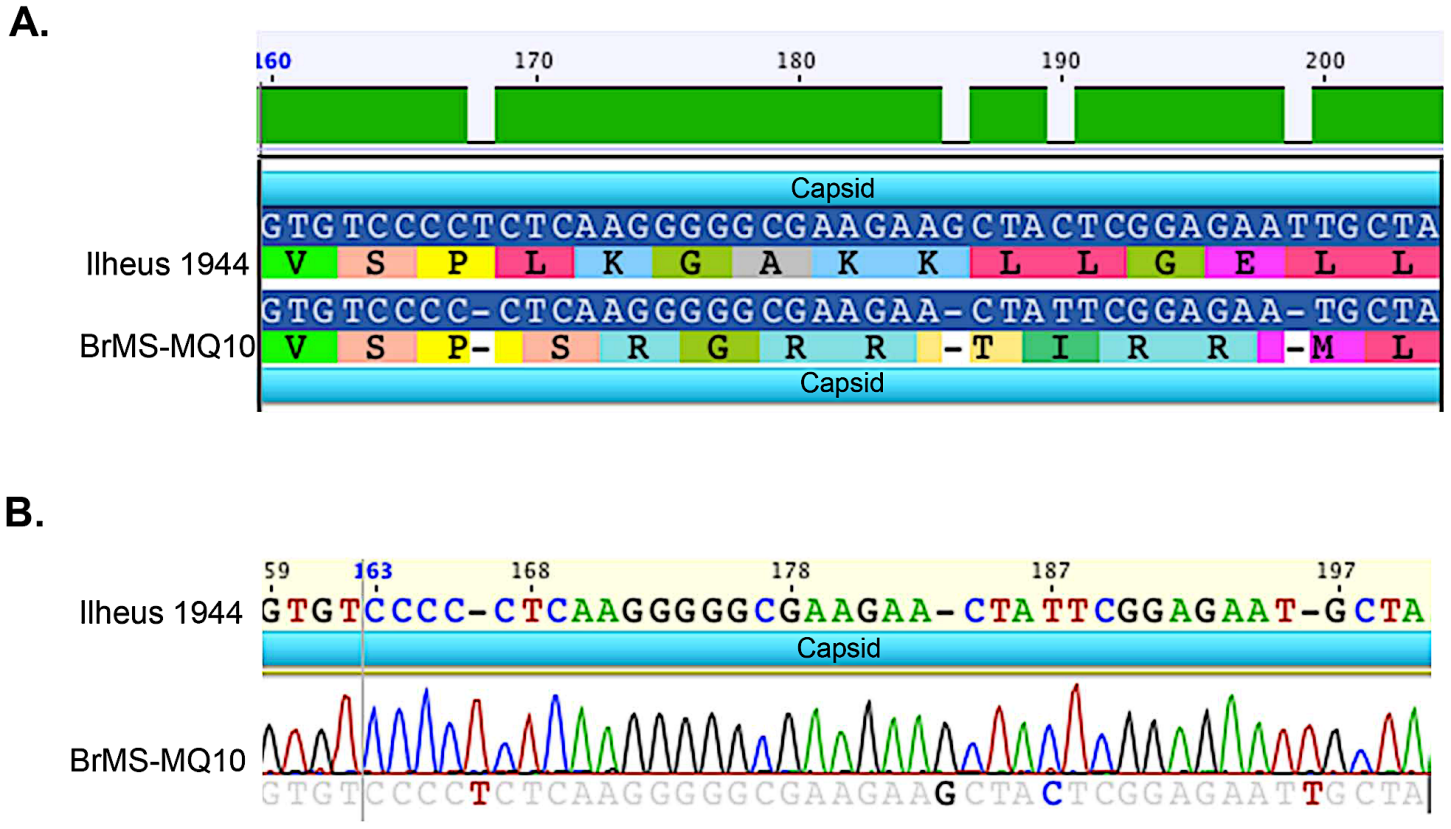
Nucleotide insertions in ILHV isolate BrMS-MQ10 as compared to available ILHV sequence. (A) Alignment between capsid amino acid 24 and 37 (nucleotides 166–204) and (B) the corresponding sequence chromatograph for the same region of BrMS-MQ10.

A total of 292 mosquito pools were assayed by cell culture and real-time RT-PCR. One pool of 50 non-engorged female *Ae. scapularis* demonstrated plaques on Vero cells and CPE in C6/36 cells on the 3^rd^ and 6^th^ day post-inoculation, respectively. A 1,034-bp cDNA amplicon was obtained, and identified as closely related to ILHV. The NCBI file with the highest identity was the Ilheus virus isolate PE163615 (GenBank: EF396950.1) and had an e-value of 0.0, a maximum score of 1688, coverage of 95%, and a maximum identity of 98%.The complete genome (10,759-bp) was sequenced and the alignment with the only full-length genome available in GenBank revealed a 95% similarity to the original strain of ILHV, isolated in Brazil in 1944 (NCBI Reference Sequence: NC_009028) (Figure 2). Phylogenetic analysis grouped the NS5 and Envelope sequences of our isolate with Ilheus virus isolate FSE800 (GenBank: EF396947.1) (Figure 3) isolated from a human case in Ecuador [11]. A total of 44 nonsynonymous amino acid changes were identified between our isolate and the GenBank Ilheus 1944 isolate. Three nucleotide inserts were identified in the capsid region creating an amino acid shift between amino acid residue number 24 and 37 (nucleotides 166–204) (Figure 4; Table 2). The genome sequence of our isolate of ILHV designated as BrMS-MQ10 was deposited in the publicly accessible database GenBank under accession number KC481679. This strain was isolated from mosquitoes landing on research team members on April 18^th^, 2010 from 5:35 to 7:00 pm in a natural drying lake near an artificial reservoir. Several additional viruses were detected by mosquito cell culture and/or real-time RT-PCR that did not align with known sequences on GenBank. These viruses will be described separately.

**Table 2:** Nucleotide and amino acid differences between IHLV 1944 and the field isolate ILHV BrMS-MQ10.

Region	Nucleotide	Amino Acid	ILHV 1944	ILHV BrMS-MQ10
5′ UTR	20	-	-	A (insert)
Capsid	168	25	-	T (insert)
	186*	31	-	G (insert)
	190*	32	T	C
	199*	35	-	T (insert)
	205	37	Asp	Ser
	208	38	Val	Gly
	211	39	Arg	Lys
	220	42	Pro	Ile
	440	116	Ala	Val
Pre	473	10	Asp	Gly
Env	1391	147	Ile	Thr
	2052	367	Lys	Asn
	2123	391	Asn	Ser
NS1	3053	200	Phe	Tyr
	3061	202	Gln	Glu
	3068	205	Thr	Lys
	3385	310	Asn	Asp
	3438	328	Glu	Asp
	3454	333	Val	Met
NS2A	3590	25	Arg	Lys
	4124	203	Ala	Val
NS3	5027	147	Thr	Ile
	5047	153	Ile	Lys
	5049	154	Ile	Lys
	5057	157	Glu	Ala
	5061	158	Arg	Ser
	5255	223	Val	Ala
	5366	260	Ala	Val
	5371	261	Arg	Cys
	6311	575	Val	Asp
	6314	576	Arg	Asn
	6318	577	Gly	Asn
	6321	578	Asp	Glu
	6324	579	Pro	Val
	6328	580	Val	Ile
NS4A	6646	68	Thr	Ala
NS4B	6947	20	Ser	Thr
	6970	27	Thr	Ala
	6978	30	Gln	His
NS5	9330	559	Asp	Glu
	9509	619	Ala	Val
	9790	712	Gly	Ser
	10162	836	Trp	Gly
	10180	842	Ile	Leu
	10310	886	Cys	Tyr
3′UTR	10383	-	C	A
	10504	-	T	C
	10552	-	-	C (insert)
	10562	-	C	T
	10645	-	G	T
	10731	-	A	T
	10754	-	T	-

* Falls in a region where inserts have offset the alignment (see figure 4).

## Discussion

In the present work we confirm ILHV activity in the Nhecolândia sub-region of Pantanal, through the isolation of ILHV from a pool of *Ae. scapularis*, which was the most prevalent mosquito species identified, and one that has transmitted ILHV efficiently in laboratory vector competence studies [35]. The anthropophilic behavior, relatively high prevalence, and susceptibility to infection (minimum infection rate observed 2.5 per 1,000) implicate *Ae. scapularis* as a suspect vector of medical importance for ILHV in the Brazilian Pantanal. This species is known for its gonotrophic discordance, a trait that amplifies its importance as a vector [36].

The isolated strain, BrMS-MQ10 had 44 nonsynonymous mutations identified as compared to the only other ILHV full genome sequence available in GenBank. Interestingly, there were three individual nucleotide insertions identified between codon 24 and 37 of the capsid region. The capsid protein is known to form secondary structures that likely indicate the protein interacts with itself during nucleocapsid assembly [37]. Similarly, capsid has been implicating with modulating cellular pathways as well as plays a role in *cis*-acting interactions with other regions of the viral genome [38], [39]. Being only the isolate of ILHV with a full genome sequence record, the importance of these changes in the capsid region are as yet undefined. Future studies should strive to characterize the differences between the two known isolates and determine the significance of insertions in that region of the capsid.

Much remains undiscovered regarding mosquito populations in the Pantanal region. Four mosquito species, including *Ma. pseudotitillans*, *Anopheles argyritarsis*, *Cx. coronator* and *Ur. lowii*, are herein reported for the first time in the Nhecolândia sub-region. The abundance of several mosquito species differed greatly compared with a study conducted in the same region in 2007 [25]. This marked fluctuation of local mosquito populations was probably due to variations in annual rainfall and may influence arbovirus transmission dynamics in the region.

Little is known about human health conditions in the Brazilian Pantanal [40]. The previously reported high prevalence of neutralizing antibodies for ILHV in horses [20] combined with our isolation of ILHV in local mosquitoes confirm the activity of an arbovirus of potential medical importance in the region. The isolation of ILHV infection in mosquitoes while attempting to blood feed on a human warrants further investigation of this virus by the Brazilian arbovirus surveillance program and local physicians.
