# Correction: Ilheus Virus Isolation in the Pantanal, West-Central Brazil

**DOI:** 10.1371/annotation/13ca0354-e5eb-42bd-b99c-606fe873df2c

**Published:** 2013-07-26

**Authors:** Alex Pauvolid-Corrêa, Joan L. Kenney, Dinair Couto-Lima, Zilca M. S. Campos, Hermann G. Schatzmayr, Rita M. R. Nogueira, Aaron C. Brault, Nicholas Komar

The graph label in Figure 4A is incorrect. The two labels, Ilheus 1944 and BrMS-MQ10, should be reversed.

